# Changes in Physical Activity and Health Indicators among Koreans during the COVID-19 Pandemic: Comparison between 2019 and 2020

**DOI:** 10.3390/healthcare10122549

**Published:** 2022-12-15

**Authors:** Kyungsik Kim, Shuai Zhang, Pan Ding, Yongdi Wang, Brain H. Yim, Zheming Hu, Sihong Sui

**Affiliations:** 1 Department of Sport & Leisure Studies, Hoseo University, Asan-si 31499, Republic of Korea; 2 College of Physical Education, Jilin Normal University, Siping 136000, China; 3 Sports and Health College, Guangzhou Sport University, Guangzhou 510075, China; 4 School of Foundations, Leadership and Administration, Kent State University, Kent, OH 44240, USA; 5 College of Physical Education, Jiujiang University, Jiujiang 332000, China

**Keywords:** COVID-19, physical activity, stress, depression, obesity, high blood pressure, diabetes

## Abstract

This study aimed to compare the changes in physical activity (PA), chronic disease, and mental health indicators of Koreans before and after the COVID-19 outbreak, using raw data from more than 400,000 representative samples from the 2019–2020 Community Health Survey by the Korea Centers for Disease Control and Prevention, and to explore the correlations among them. We used two-way ANOVA to analyze changes and differences in PA and obesity levels. We assessed the influence of gender and recurrent PA using chi-square tests for mental health status and chronic disease. Finally, we performed a correlation analysis to determine the relationships among PA days, mental health, and chronic disease. The results showed that, compared to the levels before the COVID-19 period, moderate-intensity (Days: 1.415~1.217; Time: 114.688~107.321) and high-intensity (Days: 0.798~0.671; Time: 112.866~106.110) PA significantly decreased in Koreans during the COVID-19 period, while low-intensity (Time: 60.305~61.735) PA increased. Before and during the COVID-19 period, men (18,436 (8.1%)~16,124 (7.0%)) performed PA more regularly than women (13,207 (5.8%)~9382 (4.1%)). Compared to the number of regular PA participants before the COVID-19 period, regular PA participants (male, female) decreased from 31,643 (13.8%) to 25,506 (11.1%) during the COVID-19 period. Compared with the levels before the COVID-19 period, the experience rates of stress (3.1%~2.6%), depression (0.8%~0.6%), HBP (3.0%~2.2%), and diabetes (1.2%~0.9%) significantly changed under different levels of conventional PA intervention. In addition, the obesity rate during the COVID-19 period (23.957) was higher than it was before COVID-19 (23.477). During the COVID-19 period, the PA of Koreans was greatly restricted, but low-intensity PA was maintained and increased. PA is an effective activity for maintaining mental health and for preventing and reducing chronic diseases. Recommendations for appropriate intensity or a combination of high-, moderate-, and low-intensity PA should be based on the health status of Koreans to help them maintain mental health and to reduce the risk of chronic diseases during COVID-19 social distancing.

## 1. Introduction

On 28 February 2020, the World Health Organization (WHO) raised its assessment of the global risk of the COVID-19 virus to the highest level, and countries have responded to this risk by adopting various effective isolation and COVID-19 virus infection containment measures (e.g., quarantines, school closures, and cancellations of various major sporting events and various cultural events) [1]. According to a BBC report on 7 April 2020, as of the end of March, more than 100 countries around the world had implemented full or partial lockdowns, affecting billions of citizens [2]. In Korea, after the first confirmed case of the COVID-19 virus on 20 January 2020, the infection spread nationwide. At the end of March 2020, the Korean government implemented a social-distancing policy (a) to prohibit going out, gathering, and using multiple facilities, (b) to ban the use of indoor sports facilities, (c) to close sports stadiums and parks, (d) to recommend telecommuting, and (e) to limit the business hours of restaurants and cafes. However, the implementation of this policy inevitably affected participation in physical activity (PA), the obstacle to which might have been staying home. Sang et al. [3] pointed out that lockdown policies had a negative impact on PA participation and that a sedentary lifestyle was prevalent during home confinement. Gülü and Ayyıldız [4] also reported that barriers to PA participation increased significantly in both men and women during COVID-19.

WHO reported HBP (13%), smoking (9%), diabetes (6%), and low PA (6%) as the leading causes of death worldwide [5]. Inadequate PA is linked to nearly 3 million deaths per year and to 6–10% of the incidence of significant non-communicable diseases [6]. Insufficient PA relates to obesity [7] and the risk of developing a variety of chronic diseases [8,9]. In contrast, exercise is not only effective in controlling blood sugar in people with diabetes but also reduces cardiovascular risk, reduces weight, protects bone health, and prevents metabolic disease [10]. The U.S. government and WHO recommend that adults engage in at least 150 min of moderate-intensity PA, 75 min of vigorous-intensity PA, or an equivalent combination of the two every week to promote health [11]. Regular exercise also reduces symptoms of depression and anxiety and acts as a stress regulator [12]. Various types of PA (e.g., aerobic and resistance training) have beneficial effects on the physical and mental health of humans [13,14,15]. In addition, various guidelines for social distancing, including bans on large-scale events and games, self-isolation of close contacts, restrictions on store hours, and restrictions on the number of people in private gatherings, have resulted in restrictions on the launch of economic and social activities [16]. During the COVID-19 quarantine period, PA can help prevent chronic diseases and promote health [17], but the various anti-epidemic measures proposed by governments and health authorities to curb the spread of COVID-19 are likely to reduce PA participation. In light of the chronic disease prevention guidelines proposed by WHO, this paradoxical situation poses a challenge that merits scholarly attention, particularly to ways in which COVID-19 has affected changes in PA, mental health, and chronic diseases.

Therefore, in view of the close relationship between physical activity and physical and mental health, as well as the needs of economic development, it is an urgent problem to clarify the changes and development trends of sports participation and physical and mental health indicators before and during the epidemic. Authoritative and authentic empirical evidence is particularly important.

Although some scholars have examined the relationship between PA and mental and physical health in the context of COVID-19, as well as trends and changes in PA, mental illnesses, and chronic diseases, e.g., [3,4,18,19,20,21], few have considered multivariate variables (e.g., PA, stress, depression, obesity, HBP, and diabetes). Existing studies about PA, chronic diseases, and mental illnesses in the context of COVID-19, e.g., [3,4,18,20,21], tend to be cross-sectional and to feature singular variables. In addition, few scholars have examined specific changes in high-, moderate-, and low-intensity PA since the onset of COVID-19 or the direct relationship between those changes and the incidences of mental illness and chronic disease. To fill this gap, we analyzed specific differences in PA before and during the COVID-19 outbreak in 2019 and 2020 using raw data from the 2019–2020 National Community Survey by the Korea Centers for Disease Control and Prevention (KCDC) and compared the effects of regular PA on changes in the health indicators of mental illness and chronic disease. The findings should provide evidence for the way COVID-19 has affected the PA and the physical and mental health of Koreans. Furthermore, the findings should have solid theoretical and practical implications for relevant government departments and researchers. Therefore, this study developed the following hypotheses:

**H1.**  *There will be differences in PA levels between genders before and during COVID-19.*

**H2.**  *There will be differences in stress by gender and regular PA before and during COVID-19.*

**H3.**  *There will be differences in depression by gender and regular PA before and during COVID-19.*

**H4.**  *There will be differences in obesity by gender and regular PA before and during COVID-19.*

**H5.**  *There will be differences in HBP by gender and regular PA before and during COVID-19.*

**H6.**  *There will be differences in diabetes by gender and regular PA before and during COVID-19.*

**H7.**  *The days of PA will be correlated with mental health and chronic diseases before and during COVID-19.*

## 2. Materials and Methods

### 2.1. Research Design

We first received approval from KCDC to use data from their 2019–2020 Community Health Survey to investigate changes in PA, mental health, obesity, and chronic diseases before and since the COVID-19 outbreak. KCDC has conducted the Community Health Survey, approved by the National Statistical Office, since 2008 to produce community health statistics for the establishment and evaluation of regional health and medical plans based on the Local Health Act. The survey sample included adults who were at least 19 years old and resided in cities, counties, and wards across Korea. Sampling was stratified according to administrative district unit (i.e., Dong/Eup/Myeon) and housing type. The primary sampling point was a probability-proportional systematic extraction based on the number of households in the Tong/Ban/Ri district. The secondary sampling point was a phylogenetic extraction based on the number of households in Tong/Ban/Ri. From August to October every year, after notification of household selection, KCDC investigators visit households, explain the community health survey to the subjects, obtain consent to participate, and use a computer-assisted personal interview (CAPI)—a 1:1 interview method using an electronic questionnaire—to conduct the survey [22,23]. In this study, we designated 2019 as “before COVID-19” and 2020 as “during COVID-19.” The number of samples was 229,099 before COVID-19 and 229,269 during COVID-19. Based on the enforcement regulations of the Bioethics and Safety Act (Article 2, Paragraph 2), the Community Health Survey was not subject to IRB deliberation because it did not fall under the study of human subjects [24].

### 2.2. Measures

Demographic characteristics included gender, age, education level, income level, occupation, and marital status. Age classifications were 19–29 years old, 30–39 years old, 40–49 years old, 50–59 years old, 60–69 years old, 70–79 years old, and 80 years old or older. Education levels included less than elementary school graduate, middle school graduate, high school graduate, university graduate, and graduate school or higher. Occupations included managers; experts, and related workers; office workers; service workers; sales workers; agricultural, forestry, and fishery workers; craftsmen and related workers; equipment, machine operation, and assembly workers; simple laborers; soldiers; students; housewives; and unemployed. Marital status types included having a spouse, divorced, widowed, separated, and not married.

We measured PA based on the number of days per week and the amount of exercise time per day of high-intensity PA, moderate-intensity PA, and low-intensity PA. High-intensity PA included exercises such as running (jogging), mountain climbing, high-speed biking, fast swimming, soccer, basketball, jumping rope, squash, and singles tennis. The number of days of high-intensity PA was the number of days during the past week when the participant felt very tired after exercise or engaged in vigorous PA for more than 10 min to the point of being out of breath. The time of high-intensity PA was the number of hours and minutes per day (i.e., “How many minutes per day did you usually engage in vigorous PA?”). Moderate-intensity PA included exercises such as slow swimming, doubles tennis, volleyball, and table tennis, but not walking. Questions included “In the past week, on how many days did you engage in moderate-intensity PA for 10 min or more, making you a little more breathless than usual?” and “How many hours per day did you usually engage in moderate-intensity PA?” Low-intensity PA included both moving and walking, and the questions were comparable. We calculated the regular PA practice rate (hereafter, regular PA) based on the number of people who participated in high-intensity PA for at least 20 min at least 3 days a week in the past week or the number of people who participated in moderate-intensity PA for at least 30 min at least 5 days a week [25]. In this study, we converted the exercise time to minutes before analysis. We assigned a score of “1” to regular PA cases and a score of “0” to cases that did not correspond to regular PA.

We measured mental health according to stress and depression experience rates. To indicate how much stress they felt in their daily lives, respondents chose one of the following: “I feel very little (1)”, “I feel a little bit (2)”, “I feel a lot (3)” and “I feel very much (4)”. We combined 1 and 2 into a score of “0” and 3 and 4 into a score of “1” to construct the stress-experience rate variable. For the rate of depression experienced, respondents answered “Yes” or “No” to the following question: “Have you ever felt sad or hopeless enough to interfere with your daily life for more than 2 weeks in a row in the past year?

We measured obesity rates based on Body Mass Index (BMI), which is body weight (kg)/height (m^2^). For the purpose of this study, a BMI of 18.5 or less was “underweight”, 18.5 to 22.9 was “normal”, 23.0 to 24.9 was “overweight” and 25.0 or more was “obese”. The chronic disease rate included the incidence of HBP or a diabetes diagnosis (i.e., “Have you ever been diagnosed with high blood pressure by a doctor?” and “Have you ever been diagnosed with diabetes by a doctor?”).

### 2.3. Statistical Analysis

**Figure 1:**
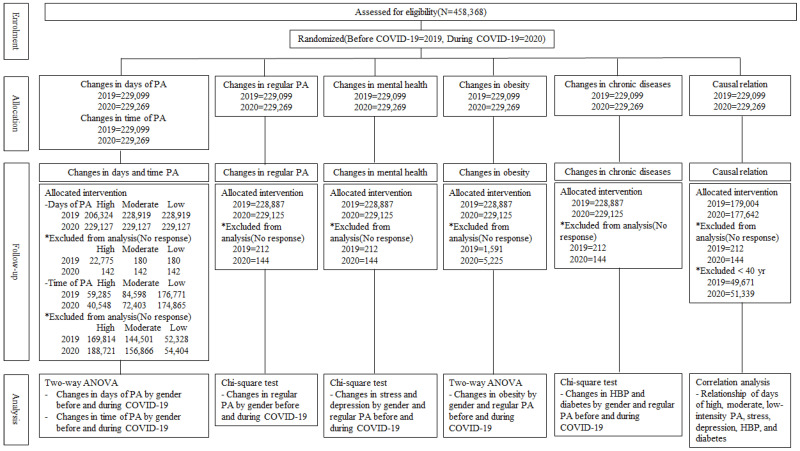
Flow diagram of data analysis.

We analyzed the data using two-way ANOVA (analysis of variance), a chi-square (χ^2^) test, and correlation analysis with an SPSSWIN 25.0 program. In present study, non-response was excluded to find out the changes in days and time of PA, regular PA, mental health, obesity, chronic health, and correlation relation by PA intensity before and during COVID-19. The size of the analysis data differs by year and PA intensity due to non-response. Details are indicated by asterisks in Figure 1. We expressed the characteristics of the participants as proportions (%). We used two-way analysis of variance to identify changes in high-, moderate-, and low-intensity PA by gender and year (before and during COVID-19), and changes in obesity by gender and regular PA (participants and non-participants) before and during COVID-19. Two-way ANOVA is a useful analysis method for verifying the interaction effects of gender and year, and gender and regular PA. That is, changes in physical activity according to gender and year and changes in obesity by gender and regular PA can be analyzed, and the interaction effect between these variables can be verified.

The present study used the chi-square test to analyze changes in regular PA by gender before and during COVID-19 and changes in mental health (stress, depression), chronic diseases (HBP, diabetes) by gender and regular PA before and during COVID-19. The chi-square test is a useful technique for verifying changes in PA, mental health, and chronic disease experience rates by gender, considering the periods before and during COVID-19. Finally, correlation analysis revealed the relationship between days of PA, mental health, obesity, and chronic diseases. We conducted a correlation analysis by converting the dichotomous variables of stress, depression, HBP, and diabetes into 1 (experience) and 0 (non-experience) variables. The detailed flow diagram of data analysis is shown in Figure 1.

In this study, Cohen’s [26] criterion was used to verify the effect size of the mean difference in ANOVA. The effect size is interpreted as a small effect size of 0.1 or less, a medium effect size of 0.3, and a large effect size of 0.5 or more based on the standardized correlation coefficient interpretation standard [26]. The statistical significance level was *p* < 0.05.

## 3. Results

### 3.1. Characteristics of Participants

Table 1 presents the characteristics of participants. Women outnumbered men both before (55.2%) and during (54.7%) COVID-19. Most participants were 50–69 years old before (38.9%) and during (39%) COVID-19, followed by people older than 70 years before (23.6%) and during (22.8%) COVID-19. The largest majority of participants were university graduates or higher before (36.1%) and during (37.6%) COVID-19. Before COVID-19, agriculture, forestry, and fisheries workers accounted for 11.1% of participants, and during COVID-19, simple workers accounted for 10.1%. Most participants had a spouse before (66.4%) and during (62.6%) COVID-19.

### 3.2. Changes in PA before and during COVID-19

**Figure 2:**
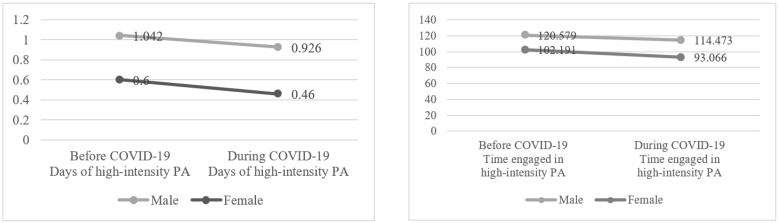
Changes in high-intensity PA by gender before and during COVID-19. Note: Time represents the number of minutes exercised per week.

Figure 2 shows the results of two-way ANOVA to compare the days and amounts of high-intensity PA by gender before and during COVID-19. These results represent the percentage of people who participated in PA more than one day per week. Men engaged in high-intensity PA more than women before (*p* < 0.001; *d* = 0.26) and during (*p* < 0.001; *d* = 0.29) COVID-19. Specifically, the number of days of high-intensity PA before and during COVID-19 significantly decreased from 1.042 to 0.926 for men (*p* < 0.001; *d* = 0.06) and from 0.600 to 0.460 for women (*p* < 0.001; *d* = 0.10; Year F = 687.086, *p* < 0.001; Gender F = 8652.905, *p* < 0.001, Year × Gender F = 5.973, *p* < 0.05). The amount of time per day of high-intensity PA before and during COVID-19 decreased significantly from 120.579 to 114.473 for men (*p* < 0.001; *d* = 0.05) and from 102.191 to 93.066 for women (*p* < 0.001; *d* = 0.09; Year F = 119.568, *p* < 0.001; Gender F = 816.258, *p* < 0.001; Year × Gender F = 4.698, *p* < 0.05). In terms of overall mean before and after COVID-19, the number of days of high-intensity PA decreased from 0.798 to 0.671 (*p* < 0.001; *d* = 0.08), and the amount of time of high-intensity PA decreased from 112.866 to 106.110 (*p* < 0.001; *d* = 0.06). All the effect sizes for the analyses were considered small [26]. The interaction effect of gender and year was also statistically significant (*p* < 0.05).

**Figure 3:**
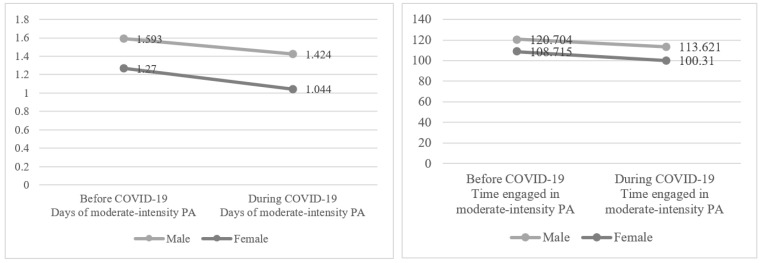
Changes in moderate-intensity PA by gender before and during COVID-19. Note: Time represents the number of minutes exercised per week.

Figure 3 shows the results of two-way ANOVA to compare the days and amounts of moderate-intensity PA by gender before (*p* < 0.001; *d* = 0.15) and during (*p* < 0.001; *d* = 0.18) COVID-19. Men engaged in moderate-intensity PA more than women before and during COVID-19. The number of days of moderate-intensity PA before and during COVID-19 significantly decreased from 1.593 to 1.424 for men (*p* < 0.001; *d* = 0.07) and from 1.270 to 1.044 for women (*p* < 0.001; *d* = 0.11; Year F = 952.305, *p* < 0.001; Gender F = 3029.707, *p* < 0.001; Year × Gender F = 20.135, *p* < 0.05). The amount of time per day of moderate-intensity PA before and during COVID-19 decreased significantly from 120.704 to 113.621 for men (*p* < 0.001; *d* = 0.06) and from 108.715 to 100.310 for women (*p* < 0.001; *d* = 0.08; Year F = 182.237, *p* < 0.001; Gender F = 486.237, *p* < 0.001; Year × Gender F = 1.329, *p* > 0.05). In terms of overall mean before and after COVID-19, the number of days of moderate-intensity PA decreased from 1.415 to 1.217 (*p* < 0.001; *d* = 0.09), and the amount of time of moderate-intensity PA decreased from 114.688 to 107.321 (*p* < 0.001; *d* = 0.07). The effect sizes were found to be small according to Cohen’s [26] convention to interpret effect size. The interaction effect of gender and year was also statistically significant (*p* < 0.001), but not for amount of time per day (*p* > 0.05).

**Figure 4:**
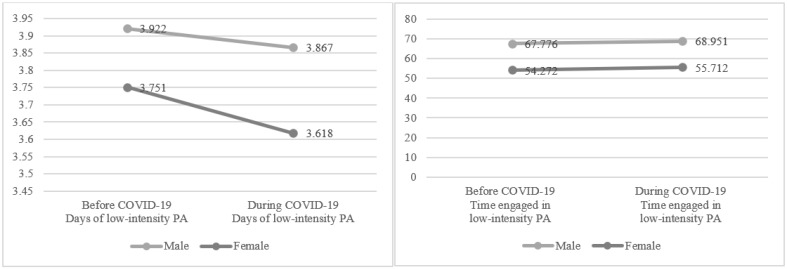
Changes in low-intensity PA by gender before and during COVID-19. Note: Time represents the number of minutes exercised per week.

Figure 4 shows the results of two-way ANOVA to compare the days and amounts of low-intensity PA by gender before (*p* < 0.001; *d* = 0.06) and during (*p* < 0.001; *d* = 0.09) COVID-19. Men engaged in low-intensity PA more than women (*p* < 0.001). The number of days of low-intensity PA before and during COVID-19 significantly decreased from 3.922 to 3.867 for men (*p* < 0.001; *d* = 0.02) and from 3.751 to 3.618 for women (*p* < 0.001; *d* = 0.05; Year F = 137.037, *p* < 0.001; Gender F = 673.777, *p* < 0.001; Year × Gender F = 23.295, *p* < 0.001). On the other hand, the amount of time per day of low-intensity PA before and during COVID-19 increased significantly from 67.776 to 68.951 for men (*p* < 0.001; *d* = 0.02) and from 54.272 to 55.712 for women (*p* < 0.001; *d* = 0.05; Year F=35.621, *p* < 0.001; Gender F = 3726.379, *p* < 0.001; Year × Gender F = 0.368, *p* > 0.05). In terms of overall mean before and after COVID-19, the number of days of low-intensity PA decreased from 3.828 to 3.731 (*p* < 0.001; *d* = 0.04), the amount of time of low-intensity PA increased from 60.305 to 61.735 (*p* < 0.001; *d* = 0.02). The results showed small effect sizes [26]. The interaction effect of gender and year was also statistically significant (*p* < 0.001), but not for amount of time per day (*p* > 0.05).

**Figure 5:**
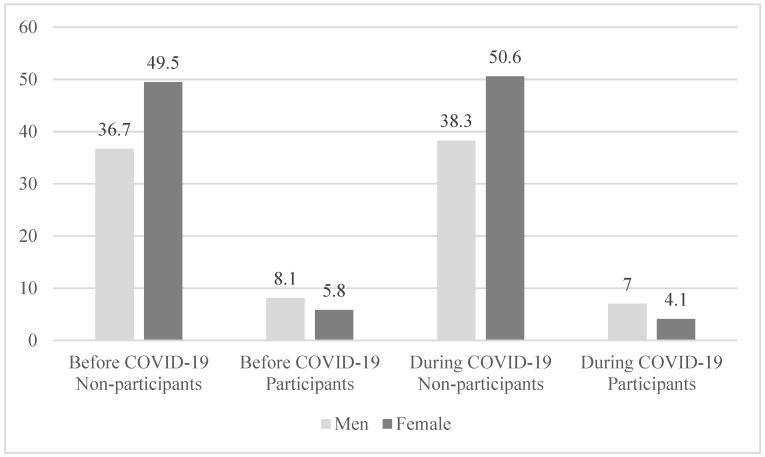
Changes in regular PA participants by gender before and during COVID-19.

Figure 5 shows the results of a chi-square test to compare regular PA by gender before and during COVID-19. While women had more regular PA non-participants than men before COVID-19, men had a higher number of regular PA participants than women. During COVID-19, similar trends appeared as before COVID-19 (*p* < 0.001). Comparing the periods before and during COVID-19, the number of regular PA particpants decreased from 18,436 (8.1%) to 16,124 (7.0%) for men and from 13,207 (5.8%) to 9382 (4.1%) for women. As a result, overall regular PA participants decreased on average from 31,643 (13.8%) to 25,506 (11.1%).

### 3.3. Changes in Mental Health before and during COVID-19

We conducted a chi-square test with gender as a control variable to observe changes in the rates of stress and depression experience according to regular PA before and during COVID-19. Table 2 shows that, for men and women, stress was lower in regular PA participants than in non-participants and higher in men than in women (*p* < 0.01, *p* < 0.001). In the case of non-participants, stress increased from 16.9% to 17.7% among men and from 20.7% to 21.0% among women. On the other hand, among regular PA participants, stress decreased from 3.9% to 3.5% for men and from 2.4% to 1.9% for women. Looking at the total results, this trend is even more clear. The rate of stress experience increased from 19.0% to 19.5% in regular PA non-participants but decreased from 3.1% to 2.6% in regular PA participants (*p* < 0.001). These results are noteworthy evidence that regular PA reduced stress during COVID-19.

As shown in Table 3, before COVID-19, the rate of depression experience by gender and regular PA was lower in physically active men and women. During COVID-19, the rate of depression experience was also lower for both men and women among regular PA participants than among non-participants. These results indicate that regular PA helped reduce depression. Ironically, in the total results, the rate of depression experience decreased from 5.4% to 5.0% among regular PA non-participants and from 0.8% to 0.6% among regular PA participants.

### 3.4. Changes in Obesity and Chronic Diseases before and during COVID-19

**Figure 6:**
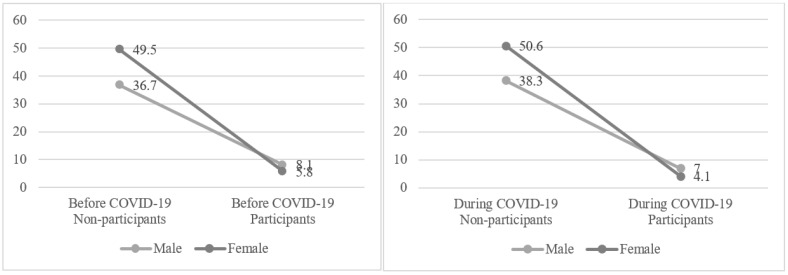
Changes in obesity by gender and regular PA before and during COVID-19. Note: Obesity rates were determined using BMI.

Figure 6 shows the results of two-way ANOVA to compare the changes in obesity by gender and regular PA before and during COVID-19. Before COVID-19, the obesity rate was higher in physically active men and women (Regular PA F = 1162.530, *p* < 0.001; Gender F = 112.085, *p* <0.01; PA × Gender = 5.898, *p* < 0.05). During COVID-19, more men than women were regular PA participants and more women than men were regular PA non-participants (Regular PA F = 4599.851, *p* < 0.001; Gender F = 20.147, *p* < 0.001; PA × Gender = 126.728, *p* < 0.001). The interaction effects of gender and regular PA were all statistically significant before and during COVID-19.

A notable result is that the obesity rate was higher for both male and female regular PA participants and non-participants during COVID-19 than before COVID-19. For example, in the case of men, obesity increased from 23.556 to 24.250 among regular PA non-participants and from 24.025 to 24.601 among regular PA participants. In terms of overall mean before and after COVID-19, obesity increased from 22.903 to 23.540 among regular PA non-participants and from 23.477 to 23.957 among regular PA participants (*p* < 0.001).

We conducted a chi-square test with gender as a control variable to observe changes in the rate of chronic disease diagnosis by regular PA before and during COVID-19. Table 4 shows that, for men and women, the rate of HBP diagnosis by gender and regular PA was lower in regular PA participants than in regular PA non-participants (*p* < 0.001). These results suggest that PA helped reduce HBP. HBP remained unchanged at 25.7% in regular PA non-participants but decreased from 3.0% to 2.2% in regular PA participants (*p* < 0.001).

As shown in Table 5, the rate of diabetes diagnosis by gender and regular PA before and during COVID-19 was significantly lower in regular PA participants than non-participants for both men and women (*p* < 0.001). These results show that PA helped prevent or reduce diabetes. Diabetes decreased from 11.6% to 10.8% in regular PA non-participants and from 1.2% to 0.9% in regular PA participants (*p* < 0.001).

### 3.5. Correlation between Number of Days of PA, Mental Health, and Chronic Diseases before and during COVID-19

We performed a correlation analysis only for people over 40 years old to investigate the relationships between the number days of PA, mental health, and chronic diseases before and during COVID-19 because the occurrence of chronic diseases tends to increase in that age group. Table 6 shows negative correlations between number of days of PA and stress, depression, HBP, and diabetes before and during COVID-19. This trend means that as the number of days of PA increased, stress and depression, as well as HBP and diabetes, decreased. These correlations held when comparing values before and during COVID-19. As PA decreased during COVID-19, the negative correlations with stress, depression, and HBP tended to be weaker than before COVID-19. However, the number of days of high- and moderate-intensity PA reinforced the negative correlation with diabetes. Excluding the relationship between the number of days of high-intensity PA and stress, the current results indicate that increasing PA improved mental health and reduced chronic disease.

## 4. Discussion

This study investigated Korean people’s participation in high-, moderate-, and low-intensity PA, mental health, including stress and depression, and physical health, such as obesity, HBP, and diabetes, before and during COVID-19. Then, participation in PA and health indicators were compared before and during COVID-19 to determine changes in the Korean health situation after the outbreak of COVID-19. Moreover, a further correlation analysis of PA and health indicators was conducted to explore the changes in Korean health after the outbreak of COVID-19, so as to provide guidance to maintain health during COVID-19.

Our findings are the first to verify changes in PA and health indicators among Koreans using a large-scale sample. We found that the COVID-19 pandemic led to notable changes in PA, mental health, obesity, and chronic diseases among Koreans. Even if Cohen’s effect size is small, the mean difference across national surveys is meaningful in discussing changes in physical activity before and during COVID-19.

Before and during COVID-19, high-intensity and moderate-intensity PA among Koreans decreased, while low-intensity PA increased. In addition, regular PA decreased. Korean adult women had lower PA levels than adult men before and during COVID-19. This finding represents a gender inequality in PA regardless of COVID-19 and shows that this trend has persisted since the outbreak. Gender inequality in sports closely relates to Korea’s traditional gender-role socialization, which emphasizes inactivity, modesty, and femininity for women. These cultural values have acted as limiting factors in women’s PA. At the same time, regular PA participation is complex and dynamic, and influenced by various factors. For instance, previous findings show that barriers to PA were significantly greater for women than men in all subdimensions, including self-perceived health status, during the COVID-19 lockdown [4]. In addition, poor health (57.7%), lack of friends (43.0%), and lack of interest (36.7%) were identified as common obstacles to PA in a study by Moschny et al. [27]. Likewise, our findings confirm that social distancing and quarantine due to COVID-19 had negative effects on the overall PA of Koreans. Indeed, physical inactivity increased globally after the COVID-19 outbreak. Dumith et al. [28] revealed that before the outbreak, approximately 20% of adults were physically inactive with a 17.4% prevalence of physical inactivity after weighting for the global population, and that after the outbreak, both numbers increased. López-Bueno et al. [29] found that weekly PA among Spanish adults reduced by 20% as a result of COVID-19 restrictions. In addition, Karuc et al. [30] found a significant decrease in moderate-to-vigorous PA among both male and female young adults.

Findings from a study about disabled Koreans by Cho et al. [19] support our finding that low-intensity PA increased while high-intensity and moderate-intensity PA decreased after the onset of COVID-19. Similar findings show that confinement due to COVID-19 caused a 16.8% decrease in self-reported high-intensity PA among 3800 Spanish adults [31] and that leisure-time activities increased among Greek adults [32]. These results are consistent with the present results; i.e., although the days of low-intensity PA decreased, the daily PA lasted longer, so the overall low-intensity PA increased when comparing the period before COVID-19 with the period during COVID-19. In Korea, the 2020 social-distancing policy reached level 2.5, which is close to level 3. At level 2.5, sporting events take place without spectators and all entertainment facilities, performance halls, and indoor sports facilities must close for an extended period. Such social distancing greatly reduced PA among Koreans, and many Koreans were more interested in and participated in low-intensity PA (e.g., walking) to maintain their health due to the closure of indoor facilities.Therefore, Hypothesis 1 is supported, concerning the differences in the PA level between genders before and during COVID-19.

Social distancing and staying at home undoubtedly helped prevent the spread of COVID-19. Nevertheless, lockdowns led to an economic downturn and losses worldwide [33], income losses [34], and less in-person communication, all of which had adverse impacts on mental health [35,36,37]. Our findings indicate that before and during COVID-19, the rate of stress experience among regular PA participants was lower than that of non-participants. At the same time, for men and women, stress increased among regular PA non-participants and decreased among regular PA participants, a trend that also characterized the total sample, which supported Hypothesis 2 concerning differences in stress by gender and regular PA before and during COVID-19. These results show that PA helped reduce stress, especially during the outbreak, confirming previous findings. For instance, Demont-Heinrich [38] found that people with lower levels of PA showed greater psychological stress than those who were more active. Kelley and Kelley [39] and Lederman [40] pointed out that PA of any intensity related to better sleep, which plays an important role in mental health [41]. More recent findings showed that PA is an important factor in maintaining mental health and combating the effects of prolonged social isolation [42]. Therefore, regular PA is likely to help relieve mental stress during social isolation due to COVID-19.

Scholars have consistently found that the fear and uncertainty due to COVID-19 have risen, inducing mental health disorders over time (e.g., depression and anxiety). For instance, recent findings noted various changes in psychological disorders among employees [43,44]. In addition, KCDC emphasized limiting the number of users and operating hours of multi-use facilities to avoid social gatherings [45], intensifying feelings of isolation and psychological anxiety among the population [46]. Huang and Zhao [47] and Pierce et al. [48] reported that prolonged social restrictions due to COVID-19 led to difficulties in school, requirements to work at home, reduced income, and higher numbers of layoffs among Koreans, who tended to suffer high levels of depression. However, we found that, both before and during COVID-19, the rate of depression experience was lower, for men and women, among regular PA participants than among non-participants. These results indicate that regular PA helped reduce depression. Many previous findings suggested that participation in moderate-intensity PA can reduce the symptoms of depression [49,50,51] brought on by the COVID-19 pandemic. Joshua [52] also found that regular PA improved the symptoms of depression. More interestingly, according to horizontal data, the rate of depression experience was lower, for both men and women, among regular PA participants and non-participants during COVID-19 than before COVID-19, which supported Hypothesis 3. Combined with the results of this study that the amount of low-intensity PA increased significantly during COVID-19 than before, it can be said that low-intensity PA as a way to relieve depression symptoms during lockdowns.

Hypothesis 4 was supported by our findings, which showed that during COVID-19, obesity rates were higher for both male and female regular PA participants and non-participants than before COVID-19. Specifically, the BMI of regular PA participants was higher before and during COVID-19, implying that overweight people participated in PA to lose weight. These results indicate an increase in the obesity rate among Koreans, likely because time at home increased and PA decreased due to social distancing. In this regard, in Korea, buzzwords, such as “Hwagjjinja” (Korean pronunciation) and “the COVID 10”, have emerged. “The COVID 10” refers to the 10 lbs. gained during the COVID-19 lockdown. As expected, reduction in PA during COVID-19 induced weight gain. Findings reported in the scientific literature were similar [4,30,53]. In addition, Marques et al. [54] emphasized that PA helped prevent obesity in men and women, suggesting that PA promotes health.

Regular PA can reduce the risk of death from a variety of causes, including an 80% reduction in cardiovascular disease, a 90% reduction in type 2 diabetes, and a 33% reduction in cancer [55]. Numerous findings show the benefits of regular PA. In the current study, both before and during COVID-19, the rate of HBP diagnosis in regular PA participants was notably lower than that of non-participants. However, HBP diagnosis of regular PA participants, both male and female, decreased during COVID-19, which supported Hypothesis 5; combined with changes in low-intensity PA before and during COVID-19, this indicated that low-intensity PA can be recommended as a method to lower HBP. As shown in previously studies, regular PA participation helped lower blood pressure. Indeed, physical exercise is an alternative to pharmacologic therapies used to reduce HBP [56]. Aerobic exercise is a traditional prescription to treat this disease [56], although moderate-intensity training programs with a frequency of three times per week seemed to lower blood pressure most effectively [57]. Bielecka-Dabrowa et al. [58] also found that low-intensity PA can lower the risk of hypertension among the elderly.

Before and during COVID-19, the rate of diabetes diagnosis in regular PA participants was significantly lower than that of non-participants. Hypothesis 6 was supported by our finding that diabetes diagnosis of regular PA participants, both male and female, decreased during COVID-19. These findings demonstrate that regular PA helped reduce the risk of diabetes. Previous findings showed that physical inactivity increased the risk of diabetes by 42%, a number recently confirmed by Kivimaki et al. [59]. Nasonov and Samsonov [60] pointed out the wide consensus that exercise benefits patients with type 2 diabetes, especially resistance and low-intensity aerobic exercises [61]. Marques et al. [54] also found that PA helped prevent type 2 diabetes in men and women. In addition, low-intensity PA can lower the risk of type 2 diabetes in middle-aged adults and the elderly [62], a finding that is consistent with the results of this study; accordingly, it can be said that low-intensity PA is an approach recommended to control the level of blood sugar.

Yoon et al. [22] reported that daily PA decreased by 46.1% during COVID-19 in the hypertensive and diabetic group, about half of that study’s sample. A decrease in PA among chronically ill patients makes treating HBP or diabetes difficult. WHO [63] recommended “following online exercise classes” as a strategy to stay active during stay-at-home measures during the COVID-19 pandemic. This method should help people with chronic diseases, as well as the general public, maintain their health through PA.

Hypothesis 7 was supported by our analysis, which revealed negative correlations between PA and stress, depression, HBP, and diabetes. These results are consistent with previous findings. Ozdemir et al. [64] found that PA alleviated depressive symptoms by having a positive impact on psychological health, well-being, and cognitive function. In addition, accessible PA (e.g., walking and jogging) can improve mood and promote physical and mental recovery, helping people cope with the challenges of the COVID-19 pandemic [65]. Little and Francois [66] demonstrated the safety and viability of high-intensity interval training (HIIT) in controlling blood pressure. Bhaskarabhatla and Birrer [67] also found that limited PA, as a consequence of the strict quarantine during COVID-19, might affect diabetes. In addition, regular PA relates to lower resting blood pressure [68,69] and a reduced risk of type 2 diabetes [70]. 

Key limitations of this study must be recognized. On the one hand, the present study only considered data from 2019 and 2020. However, with the extension of COVID-19, Korea’s quarantine policy strengthened or loosened social distancing according to the number of COVID-19 confirmed cases, which could have an effect on the levels of PA and other variables, which could not be controlled in this study. If KCDC’s community health survey data used in this study were accumulated for several more years, covering the periods before COVID-19 (2018 and 2019) and during COVID-19 (2020 and 2021), this effect could be controlled by using the accumulated number of COVID-19 confirmed cases included in the survey items as a variable.

On the other hand, the respondents before and during COVID-19 were different, which may have resulted in biased data. Assuming that the respondents of the KCDC community health survey were the same before the outbreak of COVID-19 (in 2018 and 2019) and during the outbreak of COVID-19 (in 2020 and 2021), and applying those accumulated survey results to this study, would provide more comprehensive and objective results.

## 5. Conclusions

In order to clarify the changes in PA, mental health, obesity, and chronic diseases among Koreans before and during COVID-19, we used large-scale 2019 and 2020 community health survey data. We found that high- and moderate-intensity PA decreased substantially and that low-intensity PA increased during COVID-19. Before and during COVID-19, men performed PA more regularly than women. The decrease in moderate- and high-intensity PA led to an increase in the average obesity index during COVID-19; therefore, moderate and high-intensity PA is an important way to curb obesity. In addition, regardless of COVID-19, regular PA was beneficial for maintaining mental health and preventing and reducing chronic diseases; the correlation analysis revealed the same. High-intensity exercise has beneifts for the prevention of chronic diseases, and low- and moderate-intensity exercise has benefits for mental health. High-, moderate- and low-intensity PA needs to be used in combination, which is more conducive to physical and mental health. Although PA has declined during COVID-19, PA does help improve mental health and chronic disease. Given that changes in PA among Koreans during the COVID-19 related to a series of changes in mental and physical health indicators, researchers need to formulate reasonable PA prescriptions to help Koreans maintain mental health and prevent and reduce chronic diseases. 

The results of the current study demonstrate the importance of PA for physical and mental health. Even in the case of a substantial reduction in PA during COVID-19, PA is still beneficial to reduce the prevalence of various chronic diseases and mental illnesses. Being healthy naturally boosts your own immunity, which in turn can fight the COVID-19 virus more effectively. It will be a virtuous circle. Due to the long-term nature of the COVID-19 virus, a scientific, humanized and sensible epidemic prevention policy will be a necessary way for the sustainable development of human society. Therefore, the government and health departments need to formulate effective and reasonable epidemic prevention policies that can effectively promote the smooth progress of PA, sports participation, and various sports events. For example, in response to the contradiction between the isolation policy and the necessity of PA, we can advocate a positive epidemic prevention policy such as good protection, maintaining distance, strengthening PA and sports participation, and improving physical immunity. Fully promoting the active progress of PA, sports participation and various sports events while effectively preventing epidemics can further stimulate economic development and promote social harmony and stability.

## Figures and Tables

**Table 1:** Characteristics of participants.

Variables		before COVID-19		during COVID-19	
		Frequency	%	Frequency	%
**Total**		229,099	100	229,269	100
Gender	Male	102,572	44.8	103,894	45.3
	Female	126,527	55.2	125,375	54.7
Age	Less than 20 years	23,383	10.2	26,197	11.4
	30–39 years	26,712	11.7	25,293	11
	40–49 years	35,911	15.7	35,924	15.7
	50–59 years	44,171	19.3	44,581	19.4
	60–69 years	44,941	19.6	45,037	19.6
	70–79 years	36,191	15.8	34,626	15.1
	More than 80 years	17,790	7.8	17,611	7.7
Education	Less than elementary school	54,737	23.9	50,831	22.2
	Middle school	26,369	11.5	25,616	11.2
	High school	65,466	28.6	66,570	29
	University	74,824	32.7	78,159	34.1
	More than graduate school	7703	3.4	8093	3.5
Job	Managers	3723	1.6	3548	1.5
	Experts and related workers	19,471	8.5	19,769	8.6
	Office workers	19,696	8.6	19,916	8.7
	Service workers	17,504	7.6	16,807	7.3
	Sales workers	12,579	5.5	12,294	5.4
	Agricultural, forestry, and fishery workers	25,354	11.1	22,412	9.8
	Craftsmen and related workers	9906	4.3	9978	4.4
	Equipment, machine operation, and assembly workers	11,290	4.9	10,354	4.5
	Simple laborers	21,672	9.5	23,069	10.1
	Soldiers	728	0.3	663	0.3
	Others	87,176	38.1	90,459	39.4
Marital status	Having a spouse	152,095	66.4	143,412	62.6
	Divorced	9462	4.1	6910	3
	Widowed	28,744	12.6	27,827	12.1
	Separated	3210	1.4	10,642	4.6
	Not married	35,588	15.5	40,478	17.7

**Table 2:** Changes in stress by gender and regular PA before and during COVID-19.

Variables		before COVID-19		during COVID-19		*p*-Value *
		Stress Non-Experience	Stress Experience	Stress Non-Experience	Stress Experience	
Male	PA non-participants	66,698 (65.1%)	17,356 (16.9%)	69,284 (66.7%)	18,423 (17.7%)	0.004 0.000
	PA participants	14,455 (14.1%)	3981 (3.9%)	12,490 (12.0%)	3634 (3.5%)	
Female	PA non-participants	86,971 (68.8%)	26,219 (20.7%)	89,591 (71.5%)	26,321 (21.0%)	0.535 0.000
	PA participants	10,116 (8.0%)	3091 (2.4%)	7048 (5.6%)	2334 (1.9%)	
Total	PA non-participants	153,669 (67.1%)	43,575 (19.0%)	158,875 (69.3%)	44,744 (19.5%)	0.306 0.000
	PA participants	24,571 (10.7%)	7072 (3.1%)	19,538 (8.5%)	5968 (2.6%)	

Note * The first significance level corresponds to before COVID-19, the second significance level corresponds to during COVID-19.

**Table 3:** Changes in depression by gender and regular PA before and during COVID-19.

Variables		before COVID-19		during COVID-19		*p*-Value
		Depression Non-Experience	Depression Experience	Depression Non-Experience	Depression Experience	
Male	PA non-participants	80,344 (78.4%)	3710 (3.6%)	84,176 (81.1%)	3541 (3.4%)	0.032 0.996
	PA participants	17,688 (17.3%)	748 (0.7%)	15,475 (14.9%)	649 (0.6%)	
Female	PA non-participants	104,609 (82.8%)	8581 (6.8%)	107,992 (86.2%)	7920 (6.3%)	0.714 0.000
	PA participants	12,194 (9.6%)	1013 (0.8%)	8648 (6.9%)	734 (0.6%)	
Total	PA non-participants	184,953 (80.8%)	12,291 (5.4%)	192,168 (83.9%)	11,451 (5.0%)	0.000 0.187
	PA participants	29,882 (13.1%)	1761 (0.8%)	24,123 (10.5%)	1383 (0.6%)	

Note The first *p*-value corresponds to before COVID-19, and the second *p*-value corresponds to during COVID-19.

**Table 4:** Changes in HBP by gender and regular PA before and during COVID-19.

Variables		before COVID-19		during COVID-19		*p*-Value
		HBP Non-Experience	HBP Experience	HBP Non-Experience	HBP Experience	
Male	PA non-participants	59,193 (57.8%)	24,861 (24.3%)	62,516 (60.2%)	25,191 (24.3%)	0.000 0.000
	PA participants	14,356 (14.0%)	4080 (4.0%)	12,825 (12.4%)	3299 (3.2%)	
Female	PA non-participants	79,333 (62.8%)	33,857 (26.8%)	82,243 (65.6%)	33,669 (26.9%)	0.000 0.000
	PA participants	10,465 (8.3%)	2742 (2.2%)	7587 (6.1%)	1795 (1.4%)	
Total	PA non-participants	138,526 (60.5%)	58,718 (25.7%)	144,759 (63.2%)	58,860 (25.7%)	0.000 0.000
	PA participants	24,821 (10.8%)	6822 (3.0%)	20,412 (8.9%)	5094 (2.2%)	

**Table 5:** Changes in diabetes by gender and regular PA before and during COVID-19.

Variables		before COVID-19		during COVID-19		*p*-Value
		Diabetes Non-Experience	Diabetes Experience	Diabetes Non-Experience	Diabetes Experience	
Male	PA non-participants	72,841 (71.1%)	11,213 (10.9%)	75,906 (73.1%)	11,801 (11.4%)	0.000 0.000
	PA participants	16,690 (16.3%)	1746 (1.7%)	14,716 (14.2%)	1408 (1.4%)	
Female	PA non-participants	100,601 (79.6%)	12,589 (10.0%)	102,915 (82.1%)	12,997 (10.4%)	0.000 0.000
	PA participants	12,273 (9.7%)	12,589 (0.7%)	8772 (7.0%)	610 (0.5%)	
Total	PA non-participants	112,874 (88.4%)	26,482 (11.6%)	178,821 (78.0%)	24,798 (10.8%)	0.000 0.000
	PA participants	28,963 (12.7%)	2680 (1.2%)	23,488 (10.3%)	2018 (0.9%)	

**Table 6:** Correlation analysis of the number of days of PA, mental health, obesity, and chronic diseases before and during COVID-19.

Days of PA	Mental Health		Chronic Diseases	
	Stress	Depression	HBP	Diabetes
Days of high-intensity PA	0.002 (0.011 **)	−0.012 ** (−0.006 **)	−0.060 ** (−0.056 **)	−0.035 ** (−0.037 **)
Days of moderate-intensity PA	−0.021 ** (−0.007 *)	−0.032 ** (−0.018 **)	−0.045 ** (−0.039 **)	−0.028 ** (−0.029 **)
Days of low-intensity PA	−0.044 ** (−0.044 **)	−0.026 ** (−0.023 ^**^)	−0.027 ** (−0.016 **)	−0.014 ** (−0.009 **)

Note: Coefficients mean before (during) COVID-19; * *p* < 0.01, ** *p* < 0.001.
